# Drug repurposing for Alzheimer’s disease: a Delphi consensus and stakeholder consultation

**DOI:** 10.1186/s13195-025-01895-4

**Published:** 2025-11-18

**Authors:** Anne Corbett, Janet Sultana, Kate Stych, Roger Mills, Jeff L. Cummings, Gareth Williams, Zahinoor Ismail, Maria Soto-Martin, Jacobo Mintzer, Serge Gauthier, Nigel H. Greig, Wendy Noble, Richard Killick, Mitchell K. P. Lai, Carol Routledge, Frank Walsh, Howard Fillit, Dag Aarsland, Roger Lane, Kathryn Mills, Clive Ballard

**Affiliations:** 1 College of Medicine & Health, Faculty of Life Sciences, University of Exeter, Exeter, UK; 2 Chambers-Grundy Center for Transformative Neuroscience, Department of Brain Health, Kirk Kerkorian School of Medicine, University of Nevada, Las Vegas, USA; 3 Wolfson Sensory, Pain and Regeneration Centre (SPaRC), King’s College London, London, SE1 1UL UK; 4 Departments of Psychiatry, Clinical Neurosciences, Community Health Sciences, and Pathology & Laboratory Medicine, Hotchkiss Brain Institute and O’Brien Institute of Public Health, University of Calgary, Calgary, AB Canada; 5 Department of Geriatric Medicine, Institute of Ageing, Toulouse University Hospital, University of Toulouse III, Toulouse, F-31073 France; 6 Ralph H. Johnson VA Medical Center, Medical University of South Carolina, Charleston, USA; 7 Department of Neurology and Neurosurgery, McGill University, Montreal, Canada; 8 Translational Gerontology Branch, Intramural Research Program, National Institute on Aging, National Institutes of Health, Baltimore, MD 21224 USA; 9 Centre for Healthy Brain Aging, The Maurice Wohl Clinical Neuroscience Institute, King’s College London, London, UK; 10 Department of Pharmacology, Yong Loo Lin School of Medicine, National University of Singapore, Kent RidgeRd, Singapore, 117599 Singapore; 11 Alzheimer’s Research UK, Cambridge, UK; 12 The Icahn School of Medicine at Mount Sinai, New York, NY 10029-5674 USA; 13 Alzheimer’s Drug Discovery Foundation, New York, NY10019 USA; 14 Centre for Age-Related Medicine (SESAM), Stavanger University Hospital, Stavanger, Norway; 15 Centre for Healthy Brain Ageing, Institute of Psychiatry, Psychology and Neuroscience, King’s College London, London, UK; 16 Ionis Pharmaceuticals, Carlsbad, CA 92010 USA

**Keywords:** Alzheimer’s, Repurposing, Treatment, Delphi, Consensus

## Abstract

**Background:**

Alzheimer’s disease (AD) is an escalating global challenge, with more than 40 million people affected, and this number is projected to increase to more than 100 million by 2050. While amyloid-targeting antibody treatments (lecanemab and donanemab) are a significant step forward, the benefits of these therapies remain limited. This highlights the necessity for safe and effective compounds that offer greater therapeutic benefits to the majority of individuals with or at risk of AD. Drug repurposing allows for a cost-effective, time-efficient strategy to accelerate the availability of treatments, owing to the availability of safety information.

**Method:**

This study focuses on the third iteration of the Delphi consensus programme aimed at identifying new high-priority drug candidates for repurposing in AD. An international expert panel comprising academics, clinicians and industry representatives was convened. Through a combination of anonymized drug nominations, systemic evidence reviews, iterative consensus rankings, and lay advisory inputs, drug candidates were evaluated and ranked based on rational, non-clinical, and clinical evidence and overall safety profiles.

**Results:**

Among the 80 candidates that were nominated by the expert panel, seven underwent review, with only three candidates meeting the following consensus criteria of relevant mechanisms for targeting neurodegenerative pathways, non-clinical efficacy, and tolerability in older individuals. The three agents were: [1] the live attenuated herpes zoster (HZ) vaccine (Zostavax) [2], sildenafil, a phosphodiesterase-5 (PDE-5) inhibitor, and [3] riluzole, a glutamate antagonist. The HZ vaccine additionally offers potential for population-level dementia risk reduction.

**Conclusion:**

This Delphi consensus identified three high-priority drug repurposing candidates for AD with favourable safety profiles and mechanistic plausibility, which are considered suitable for pragmatic clinical trials, including remote or hybrid designs. The PROTECT platform, which supports international cohorts in the UK, Norway, and Canada, offers a well-established means to conduct such trials effectively, thus helping to accelerate the evaluation and potential deployment of these drug candidates to benefit individuals with or at risk for AD.

**Supplementary Information:**

The online version contains supplementary material available at 10.1186/s13195-025-01895-4.

## Background

The global challenge of dementia needs to be addressed. Over 40 million people have Alzheimer’s disease (AD), the number one cause of dementia worldwide, and this number will increase to more than 100 million by 2050 [1]. AD is a devastating neurodegenerative disease that has significant personal, financial, and societal impacts, with an estimated annual worldwide cost of more than US$800 billion.

The therapeutic benefits of cholinesterase inhibitors and memantine in clinical AD are symptomatic in a minority of people, temporary, and modest at best. Promising results have emerged from trials of the amyloid-targeting antibodies lecanemab and donanemab, with both treatments now licensed internationally [2, 3]. This is a substantial step forward and may drive better diagnostic practice by providing clinicians with a more promising treatment options. However, these disease-modifying treatments still confer only modest benefits to some, require complex protocols for administration and monitoring, are associated with significant side-effects such as Amyloid-Related Imaging Abnormalities (ARIA) and are likely to be available to only a very small number of patients. In parallel with creating effective treatment pathways to administer these treatments and developing more effective anti-amyloid disease- targeted therapies, there is an urgent need for safe and effective compounds that can provide improved therapeutic benefits to most people with or at risk of developing AD. Our increasing understanding of the broad range of potential therapeutic targets includes but is not limited to tau and amyloid pathology, neuroinflammation, synaptic dysfunction, mitochondrial dysfunction, neurogenesis abnormalities, and neuroprotection from neurodegeneration [4].

With the licensing of the new generation of disease-targeted treatments, the drug development pipeline is newly reinvigorated. However, the timeline from bench to bedside remains lengthy. Drug repurposing offers a means of fast-tracking potential new treatments to complement traditional drug discovery, largely because of established safety. Drug repurposing can be defined as “the application of approved drug compounds to new therapeutic indications” [5] and offers a route to identifying new treatments that are accessible to universities, research council programmes, and charities, complementing the work of the pharmaceutical and biotechnology sector. Trials of repurposing agents provide a platform for innovation including development of new clinical outcome measures, biomarkers, recruitment strategies, and trail design and analysis approaches [6]. Repurposing potentially offers an attractive approach to enhance traditional drug development to accelerate access to new treatments for AD and mild cognitive impairment (MCI) due to AD in the clinic.

Historically, drug repurposing has identified successful therapies across many therapeutic areas, including thalidomide which was repositioned to treat leprosy and multiple myeloma, and amantadine which was repositioned as a therapy for Parkinson’s Disease [7, 8]. An important benefit of repurposing is that the safety profile of the compound has already been determined, removing the need for additional non-clinical safety testing and toxicology studies provided the agent is used within licensed dosages. This substantially reduces the time and cost of moving treatments forward into clinical trials. Several potential approaches have been used to identify candidates for repurposing, including high-throughput screens, transcriptomic approaches, and literature reviews. Our approach, which was successfully completed in two previous iterations in 2012 and 2020, involves a combination of expert recommendation, systematic review, and Delphi consensus methodology to prioritise the best candidates [9, 10]. This approach has led to the identification of several high-priority drug candidates that are now in phase II and III clinical trials, including liraglutide (ISRCTN89711766) which is in phase III trial following a successful phase II trial [11], and fasudil (NCT06362707) and phenserine (NCT06774261) which are in phase II trials. This study describes the third series of Delphi consensus programs to identify new high-priority candidates for repurposing in AD.

## Methods

### Study design

This is a Delphi consensus study conducted in accordance with the Declaration of Helsinki. This study does not include any research participants and so does not require approval from a Research Ethics Committee. Human Ethics and Consent to Participate declarations: not applicable.

### Expert panel identification

An international expert panel was convened to deliver the Delphi consensus using our established successful methodology from previous programmes. Potential panellists fulfilled the criteria for eligibility if they were published academics and/or clinicians or industry representatives working in the field of AD, including neuroscience, neurology, psychiatry, gerontology or related fields. All panel members were invited to take part by email and participated on the basis of a specific terms of reference. An added novel element in this programme was the addition of a parallel lay advisory group comprising individuals with a lived experience of caring for someone with dementia.

### Drug candidate nominations

The Delphi panel members anonymously nominated drug candidates for consideration in the first consensus round. The list of nominated candidates was triaged to remove any already in phase 3 trials in AD and to remove analogues or closely related candidates. Candidates that received three or more nominations were taken forward for review. Throughout the process, expert contributors’ identities were anonymised, and specific feedback was sought from panellists on an individual basis through virtual reviews conducted by email.

### Systematic reviews

A systematic review of the non-clinical, clinical, and epidemiological evidence concerning the shortlisted drug candidates was carried out via predefined queries in four databases: Medline, Cochrane, PsychINFO and SCOPUS. This review was aimed at synthesising evidence concerning (i) the putative mechanism of drug action in AD; (ii) the therapeutic effect of the drug in vitro, in animal models of AD or in humans; and (iii) the safety of the drug. Systematic reviews were supplemented with highlighted key factors such as likely blood‒brain barrier penetration capability, current licensing status, safety data, posology and routes of administration.

### Iterative Delphi rounds

The systematic reviews for all longlisted candidates were circulated to the expert panel by email. The panel ranked the candidates in order of priority based on the strength of evidence via a structured feedback form. If deemed necessary, expert contributors would have undertaken subsequent ranking and consensus rounds, culminating in attending a group discussion. The objective of this process was to reach a consensus-ranked list of candidates.

### Consensus criteria and analysis of consensus

Quantitative analysis of the candidate ranking metrics was used as a means to identify consensus by calculating the median score of each candidate. Mean, median and standard deviation of all scores were calculated. A threshold of 1.75 standard deviation separation between the highest priority candidates was set as a stop/go criterion for a further round of consensus work. Descriptive justifications for each candidate were collated to support the final shortlisting process.

### Stability of results

The shortlist of highest priority candidates was circulated to the expert panel to confirm the consensus and stability of the ranking process.

### Stakeholder consultation

The final shortlist of the highest three priority candidates was prepared for lay review. Accessible summaries and infographic designs were prepared and circulated to the lay advisory group, which consisted of six members representing a diverse range of sex, age, ethnicity and employment. All the participants had experience caring for a person with dementia. The lay advisory group first completed an anonymous online survey to capture their preferences for the shortlisted candidates through a ranking process, followed by qualitative data capture of their justification for their ranking. A group discussion was then convened for the lay advisory group to discuss their opinions further, focusing on patient acceptability, perceived benefits and risks, and queries about safety and efficacy queries from the panel. At the close of the discussion, the group performed a final ranking exercise of the shortlisted candidates.

## Results

### Delphi expert panel

A total of 31 experts on dementia with experience in the pharmaceutical industry, academia and clinical setting were invited to participate in the consensus study. Of these, 28 responded, and 23 agreed to participate. Twenty-one members of the panel suggested drug candidates for consideration.

### Candidate nominations and shortlisting

**Fig. 1:**
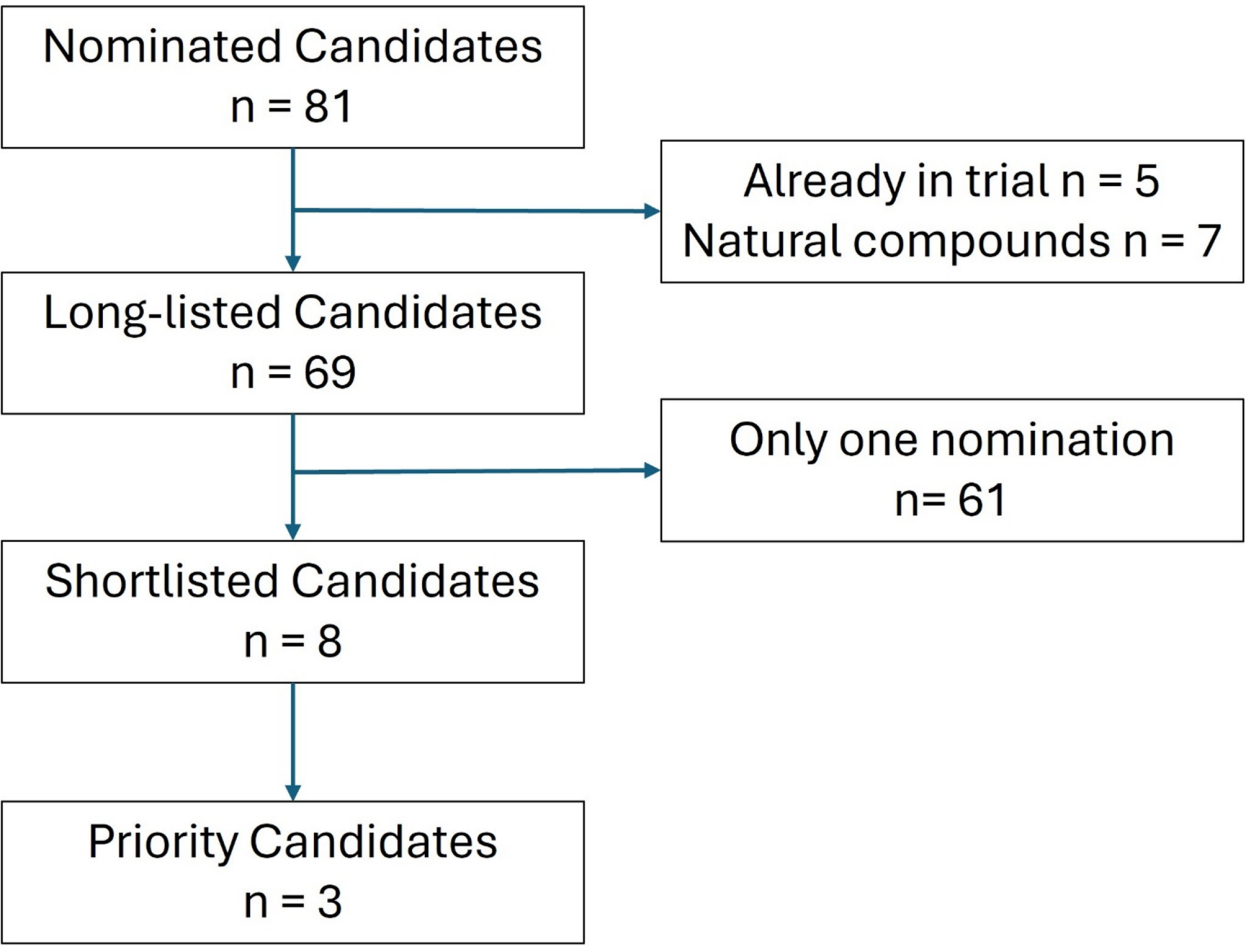
Triage of candidate treatments through the Delphi process

Overall, 80 drug candidates were nominated by the panel, seven of which were initially taken forward to the review stage following triage for duplications, candidates in existing trials and the removal of ineligible candidates (Supplementary Table 1). Following a suggestion from panel members because of new emerging literature, an eighth candidate was added (Fig. 1; Table 1). Three candidates fulfilled the consensus criteria for shortlisting after the first round of ranking, each showing a median rank score of 2 (SD 1.7), with the nearest closest candidate having a median rank of 4 (Table 2). The three lead candidates were also taken forward for review and prioritization by the lay panel (Table 3).

**Table 1:** Literature Search Results for Shortlisted Drug Candidates. Number of studies identified across databases and those included in the review

	Studies identified and screened			Total no. of studies identified and screened	No. of studies included
	MedLine	PsychINFO	Scopus		
**Sildenafil**	90	30	107	227	27
**Riluzole**	124	23	117	264	20
**Vortioxetine**	142	69	135	346	10
**Shingles vaccine**	5	0	1	6	4
**Micro-lithium**	30	17	49	96	18
**Fingolimod**	100	21	93	214	25
**Dasatinib**	10	7	27	44	5
**Cytisine**	38	7	37	82	6

**Table 2:** Compounds shortlisted by the Delphi panel

Drug Class	Proposed Candidates	Putative mechanism of action	Summary of evidence	Reason for failure to be prioritised	Rank Median (SD)
Sphingosine l-phosphate receptor modulators	Fingolimod	• Rescue of the ceramide/sphingomyelin pathway and improvement of synaptic plasticity [12]. • Increase in BDNF [13, 14]. • Modulation of glutamatergic neurotransmission ([15]. • Increase in synaptic protein abundance and by increasing post-synaptic density [16]. • Reduction of neuroinflammation and reduction of phosphorylated tau and APP levels in the hippocampus and cortex [17]. • Rescue of abnormal glial histone acetylation [18]. • Rescue of aberrant apoptotic pathways though bcl2 [19]. • Decrease in astrocyte activation [20]. • Decrease in plaque density and soluble and insoluble Aβ levels [21]. • Modulation of gamma secretase metabolism [22]. • Modulation of Akt/mTor pathway [23].	Several studies in mouse models of AD reported improved cognition with fingolimod, but evidence was not all consistent. There are no epidemiological or clinical studies.	• There was inconsistent pre-clinical evidence on benefit of fingolimod in AD and a lack of clinical studies. • Fingolimod is also unlikely to significantly cross the blood brain barrier.	3 (2.05)
Serotonin modulator	Vortioxetine	• Increase in PSD95, SYP and SYT1 expression [24]. • Prevention of the over-expression of iNOS and NADPH oxidase 2 induced by Aβ oligomers, and rescue of the mRNA expression of glutathione peroxidase 1 antioxidant enzyme; prevention of Aβ-induced neurodegeneration [25]. • Rescued of hippocampal TGF-β1 levels and synaptophysin and PSD-95) [26]. • Increase in the hippocampal expression of several neuroplasticity-related genes in middle-aged mice, such as Nfkb1, Fos, Fmr1, Camk2a, Arc, Shank1, Nlgn2, and Rab3a [27].	Vortioxetine consistently rescued cognition in mouse models of AD. It improved cognition in one study among persons with MCI but in two other studies, it was combined with cognitive training so the effect of vortioxetine itself was not known.	• Overall, there is very little evidence on the potential benefit of this drug in AD.	3 (1.64)
Mood stabiliser	Micro-Lithium	• Decrease in the burden of Aβ plaque and phosphorylated tau [28]. • Inhibition of GSK3 activity [29]. • Stimulation of neurogenesis [30]. • Decrease in cerebral oxidative and nitrosative stress markers and reduced production of pro-inflammatory; reduced expression of microglia surface receptor Trem2 and reduction in microglia recruitment towards Aβ-burdened neurons [31]. • Reduction of IL-1α, IL-6 and MIP-1B/CCL-4 gene expression and increase in IL-10 gene expression [32]. • Reduction in the activation of NFkB and inflammatory cytokines densities [32].	Lithium improved cognition in several mouse model of AD. Clinical studies reported contrasting evidence, with two suggesting that lithium did not improve cognition and two suggesting that it prevented the deterioration of cognition. There were no epidemiological studies.	• Has a very narrow therapeutic index. • There is very little evidence on the unlicensed sub-therapeutic dose, i.e., on micro-lithium itself.	4 (1.30)
Tyrosine kinase inhibitor	Dasatinib	• Inhibition of EphA4, leading to a reduction of amyloid precursor protein (APP) in cells expressing Aβ [33]. • Decrease in protein phosphotyrosine, active Src, reactive microglia and TNFα levels in APP/PS1 mice [34, 35].	One pre-clinical study found that dasatinib improved cognition. In humans, dasatinib was studied in combination with another drug, quercetin, so the therapeutic effect of dasatinib is unknown.	• Unlikely to cross the BBB. • Targets very few pathways in AD. • As a chemotherapy drug, its safety profile is not favourable for elderly persons. It is associated with a risk of myelosuppression and several other serious ADRs.	5 (1.72)
Nicotine receptor agonist	Cytisine	• Inhibition of amyloid fibril formation by the prevention α-synuclein seeding in cell assays [36]. • Increased in the release of soluble APP [37].	There is some evidence on the pharmacological action cytisine on AD pathology, but only on study on cognition in mice which showed that this drug did not lead to statistically better cognitive performance compared to other drugs (**Carrasco et al.**,** 2006**).	• Overall limited evidence. • Targets very few pathways in AD.	5 (1.96)

*Abbreviations*: *AD *Alzheimer’s disease, *ADRs *Adverse drug reactions, *APP *Amyloid precursor protein; BBB: Blood brain barrier, *BDNF *Brain-derived neurotrophic factor, *MCI *Mild cognitive impairment, *iNOS *Nitric oxide synthase, *PS *Presenilin, *TGF-β1 *Transforming growth factor β1, *TNFα *Tumour necrosis factor α

**Table 3:** Priority compounds identified by the Delphi panel and prioritised by stakeholder consultation

Drug Class	Proposed Candidates	Putative mechanism of action	Summary of evidence	Remaining work required	Rank Median (SD)	Stakeholder Rank
Vaccine	Herpes Zoster Vaccination	• Potentially on and off target mechanisms by reducing Herpes Zoster and Herpes simplex [38] • Increasing systemic immune regulation by boosting antiviral cytokine responses [39] • Hypothesised to affect the dementia disease process through a pathogen-independent immune mechanism [39] • Off target mechanisms related to enhanced immune function [40]	• Systematic review of 5 studies, with almost a million participants suggested a relative reduction of 16% in incident dementia [41] • In subsequent studies, a 20% relative reduction and 3.5% point reduction in the probability of new dementia diagnoses compared to unvaccinated individuals [42], with consistent findings from another recent report [43] • Most evidence is with the active vaccine, but a recent study did suggest a more modest reduction in dementia risk with the recombinant vaccine [44]	Phase II and III clinical trials are needed.	2 (1.70)	1
Phosphodiesterase inhibitor	Sildenafil	• Increase in neurite growth [45]. • Reduction in tau hyperphosphorylation [45–47]. • Improvement in central nervous system haemodynamic function and increase in oxygen levels [48]. • Reduction of hippocampal Aβ42 levels and in GFAP expression [49]. • Reduction in α-synuclein levels and oxidative stress [50]. • Rescue of PKG/pCREB signalling [51]. • Decrease in GSK3β and CDK5 activity and increased BDNF and Arc levels [47]. • Increase in levels of activated JNK (p-JNK) [52]and up-regulated heme oxygenase-1 [53]. • Regulation of NO-cGMP signalling [54]. • Down-regulation of pro-apoptotic proteins in aged mice [55].	• Epidemiological studies reporting contrasting findings on the protective effect of sildenafil on AD. • One small open study of sildenafil in 8 patients with AD using a novel MRI technique to examine cerebral oxygen metabolism demonstrated a significant improvement in cerebral haemodynamic function with sildenafil treatment [48]. • A further small MRI study in 10 AD patients suggests that sildenafil appears to normalize spontaneous neural activity [56] • No randomized clinical trials have been conducted.	Phase IIb and III clinical trials are needed.	2 (1.74)	2
Glutamate antagonist	Riluzole	• Inhibition of glutamatergic neurotransmission and of voltage dependent sodium channels [57]. • Protection of neuronal firing from amyloid [58] [59]) and potentiation of postsynaptic GABA receptor function (Yang et al., 2021). • Increase in BDNF levels [60]. • Normalisation of EAAT3 expression [61, 62] and of glucose metabolism [63]. • Reduction of tau [64] and Aβ plaque burden [65]. • Reduction of hippocampal AChE activity and of oxidative stress [66]. • Reduction in levels of disease-associated microglia [65]. Increase in dendrite density [67].	• Pre-clinical evidence in mice is in broad agreement that riluzole improves cognition in various mouse models of AD. • Epidemiological studies reporting contrasting findings on the protective effect of riluzole on AD. • One phase IIa 6 month clinical trial in 50 people with probable AD (MMSE 19–27) reported that riluzole had a protective effect on brain glucose metabolism compared to placebo [68]. Although underpowered for statistical evaluation, there were also numerical benefits on cognitive outcomes. • Trials in ALS, another neurodegenerative condition, show consistent benefits (Miller)	Phase IIb and III clinical trials are needed.	2 (1.72)	3

*Abbreviations*: *Arc *Activity-regulated cytoskeletal-associated protein, *BDNF *Brain-derived neurotrophic factor, *CDK5 a*nd of cyclin-dependent kinase 5, *cGMP *cyclic guanosine 3’,5’-cyclic monophosphate, *EEAT 3 *Excitatory amino acid transporter 3, *GABA *Gamma-aminobutyric acid, *GFAP *Glial fibrillary acidic protein, *GSK3β *Glycogen synthase kinase 3β, *NO *Nitric oxide

### Shortlisted candidates

#### Fingolimod

Fingolimod is a sphingosine 1-phosphate receptor modulator indicated in highly active relapsing-remitting multiple sclerosis. It could act in AD in several ways, for example, by rescuing AD pathology through the ceramide/sphingomyelin pathway and by improving synaptic plasticity [12], increasing brain-derived neurotrophic factor (BDNF) [13, 14]) and modulating glutamatergic neurotransmission [15]. It may also increase synaptic protein abundance and increase postsynaptic density [16]. Other mechanisms by which it may act include a reduction in neuroinflammation (less ramified microglia and an improved cytokine profile), reduction in phosphorylated tau and amyloid precursor protein (APP) levels in the hippocampus and cortex [17], rescue of abnormal glial histone acetylation [18] and rescue of aberrant apoptotic pathways through B-cell lymphoma 2 (bcl2) [19]. In mouse models of AD fingolimod decreased astrocyte activation, plaque density and amyloid levels in a dose-dependent manner, and evidence suggests these effects may be more pronounced once AD pathology has progressed rather than in earlier phases [20, 21, 69]. Fingolimod may also affect gamma secretase activity [22]. Protein kinase B/mammalian target of rapamycin (Akt/mTor) may be involved, as shown in a 5xFAD mouse model [23]. Although cognition in mouse models of AD improved with fingolimod, this improvement was not consistent [70]. Despite several promising non-clinical studies, no epidemiological or clinical studies have investigated the benefit of fingolimod in patients with AD. Fingolimod is unlikely to significantly cross the blood–brain barrier, which limits its potential as a repurposed drug for AD.

#### Vortioxetine

Vortioxetine is a serotonin modulator and stimulator indicated in major depressive disorder. In 5xFAD mice vortioxetine is associated with increased expression of postsynaptic density protein 95 (PSD95), synaptophysin (SYP), and synaptotagmin-1 (SYT1), but not Aβ [24]. It also prevents overexpression of inducible nitric oxide synthase (iNOS) and NADPH oxidase 2 (Nox2), rescues levels of hippocampal transforming growth factor (TGF- β1) and normalises SYP and PSD-95 levels in Aβ-injected mice [26], and rescues mRNA expression of the glutathione peroxidase 1 (Gpx1) antioxidant enzyme [25]. It increased the hippocampal expression of nuclear factor kappa B subunit 1 (Nfkb1) Fos, fragile X messenger ribonucleoprotein 1 (Fmr1), Camk2a (calcium/calmodulin-dependent protein kinase II), activity-regulated cytoskeleton-associated protein (Arc), SH3 and multiple ankyrin repeat domains protein 1 (Shank1), neuroligin 2 (Nlgn2) Ras-related in brain 3a (Rab3a)in middle-aged mice [27], all of which are related to neuroplasticity.

The effects of vortioxetine have been studied in several mouse models of AD and rescued cognition in all studies [24–27, 71–73]. Vortioxetine and cognitive training significantly improved global cognitive performance after 12 weeks in 100 adults with age-related cognitive decline in a randomised, placebo-controlled study when given in combination with cognitive training [74]. Vortioxetine in combination with cognitive training was also evaluated in another trial, but cognition was not explored as an outcome [75]. In both trials, the combined intervention consisting of drugs and cognitive training complicated analysis of the benefit of the drug itself. In a single-arm, open-label study in 111 adults with mild cognitive impairment (MCI) showed significant improvements in the Montreal cognitive assessment (MoCA), digital symbol substitution test (DSST), and clinical dementia rating (CDR) following six months of vortioxetine treatment (5–10 mg/day), with 89.6% showing reduced disease severity. Vortioxetine has a favourable safety profile [76]. There are no epidemiological studies or ongoing trials on this drug in the context of AD. The overall scarcity of evidence for this drug limits its potential for repurposing.

#### Microlithium

The term microlithium refers to very low-dose lithium. Lithium is primarily indicated in bipolar disorder. Low-dose lithium is not itself a licenced medicine. The mechanism of action of lithium is not well understood, but there are several putative mechanisms by which this drug can affect AD. Low-dose lithium decreases Aβ burden and phosphorylated tau, and inhibits glycogen synthetase kinase 3β (GSK3β) activity in mouse models of AD [28, 29]. In transgenic rats studies have reported lowering of cerebral oxidative and nitrosative stress markers (protein-bound 4-hydroxynonenal and protein-resident 3-nitrotyrosine), pro-inflammatory cytokines and microglial surface receptor Trem2 (triggering receptor expressed on myeloid cells 2) [31]. This is associated with reductions in microglial recruitment to amyloid pathology in the hippocampal CA1 region [31]. In a mouse model that mimics accelerated senescence 20 µM lithium carbonate led to reduced interleukin IL-1α (IL-α), IL-6 and macrophage inflammatory protein-1b/chemokine (C-C motif) ligand 4 (MIP-1B/CCL-4) gene expression and increased IL-10 expression [32]. Both 2 µM and 20 µM doses lead to reductions in NFkB and the density of inflammatory cytokines [32]. Microlithiuim may stimulate neurogenesis in prodromal AD [30]. One clinical study in 113 AD patients revealed that compared with placebo, microlithium is beneficial for cognition [77], although cerebrospinal fluid levels of Aβ1‒42 increased [78]. Another study reported that therapeutic doses of lithium did not rescue cognition [79]. In addition to the contrasting evidence on the benefit of microlithium, this drug has a narrow therapeutic index, and the resulting safety concerns do not make this drug a good candidate for repurposing in older individuals.

#### Dasitinib

Dasatinib is a multi-kinase inhibitor indicated in leukemia. It inhibits EphA4 (ephrin type-A receptor 4), leading to a reduction in APP in cells expressing Aβ [33]. It decreases phosphotyrosine, active Src, reactive microglia and TNFα (tumour necrosis factor-a) levels in APP/PS1 mice [34, 35]. Compared with other more promising candidates for repurposing, this drug targets only a few possible pathways in AD. In addition, it is predicted to cross the blood–brain barrier poorly. It is in multiple trials combined with quercetin as part of a “senolytic” therapy combination [80] (Gonzales, 2023).

#### Cytisine

Cytisine is a plant alkaloid high affinity partial agonist of the α4β2 nicotinic acetylcholine receptor (nAChR), and low affinity full agonist of the α7 nAChR, which is indicated for smoking cessation [81]. Cytisine targets several points in the pathogenesis of AD. It inhibits Aβ fibril formation by preventing α-synuclein seeding in cell assays [36] and increased the release of soluble APP in vitro [37]. In the secretory pathway, APP is cleaved within the Aβ domain by α-secretase to generate soluble forms of APP and p3, thus preventing the formation of Aβ. While Aβ peptides are neurotoxic, sAPPα is thought to have neuroprotective effects by modulating neuronal excitability, synaptic plasticity, neurite outgrowth, synaptogenesis, and cell survival [37]. There are no clinical trials investigating the efficacy of cytisine in AD, although two clinical studies explored the effect of cytisine on the biochemistry of AD, which reached the conclusion that selective nicotinic receptor agonists, such as cytisine, not only reduce Aβ levels but may also protect against nAChR loss in the Alzheimer’s brain [82, 83].

### Priority candidates

#### Herpes booster vaccination

Zostavax is a live attenuated form of zoster virus used to prevent herpes zoster (shingles). Developed by Merc & Co., it was approved by the U.S. Food and Drug Administration (FDA) in 2006 for adults aged 60 and older. Zostavax marked a significant advancement in preventive care for older adults, offering a strategy to reduce the severity and incidence of herpes zoster [84, 85]. The efficacy of the zoster vaccine was demonstrated in a randomised placebo-controlled trial, which revealed that Zostavax reduced the incidence of herpes zoster by 51.3% and postherpetic neuralgia by 66.5% [84]. An additional follow-up study showed the attenuation of efficacy over time but still demonstrated that Zostavax continued to provide partial protection against herpes zoster in adults [85].

Zostavax contains a viral concentration of the Oka/Merck strain of VZV (varicella-zoster virus) that is at least 14-fold greater than that of Varivax (chicken pox vaccine), inducing a stronger immune response in the elderly [86]. It is administered as a single subcutaneous dose and has generally been well tolerated. The most common adverse effects reported in clinical trials are swelling or pain at the injection site and headaches [87]. During primary VZV infection, the innate immune system rapidly responds, with interferon-alpha (IFN-α) being produced [88]. The zoster vaccine functions by boosting VZV-specific cell-mediated immunity, which helps the body control the reactivation of the virus [89]. One of its key responses is the production of IFN-α, which can block VZV replication in laboratory studies [88]. This antiviral effect plays a crucial role in reducing viral spread during primary infection and may contribute to the protective effects of the vaccine.

There are several proposed potential mechanisms relevant to AD and dementia more broadly related to zoster vaccination. As VZV is potentially associated with an increased risk of dementia, the zoster vaccine may help reduce the risk by preventing VZV reactivation directly. There are, however, also a number of other potential mechanisms, including preventing the reactivation of quiescent herpes simplex virus [38]. For the active vaccine, there may be indirect effects mediated through a pathogen-independent immune mechanism and to increased systemic immune regulation by enhancing antiviral cytokine responses [39] and amplifyinginnate immunity and heterologous T-cell immunity [40].

Recent large epidemiological studies examining vaccination for herpes zoster, mostly using the active vaccine, show significant reduction in the incidence of dementia, including AD. A systematic review of five epidemiological studies, including a total of 941,000 vaccinated individuals, estimated a significant 16% reduction in the risk of incident dementia [41, 90–93]. A series of natural experiment studies have examined the impact of vaccination on dementia incidence utilising robust methodology with careful age-matching, in the UK, Canada, New Zealand and Australia. One study in 249,000 people using UK primary care data demonstrated a 20% relative reduction and a 3.5% point reduction in the probability of new dementia diagnoses compared to unvaccinated individuals [42]. A study in 101,219 individuals in Australia demonstrated a 1.8% point reduction using the same methodology [43]. A second UK cohort study with a 6-year follow -up in 103,000 people reported outcomes with the recombinant vaccine, showing a significant 7% reduction in composite risk of dementia or death [44]. Whilst this suggests that both the active vaccine and the recombinant vaccine may be associated with a reduced risk of incident AD, it leaves open the question as to whether the effect size is potentially more substantial with the active vaccine.

#### Sildenafil

Sildenafil is a phosphodiesterase 5 (PDE 5) inhibitor that is licenced in the US, Europe and the UK for the management of pulmonary hypertension and erectile dysfunction. It was tested in the 1980 s as a treatment for angina because of its vasodilatory properties, and it entered clinical trials in 1991 but did not produce promising results [94]. It entered clinical trials again in 1993, was repurposed for the treatment of erectile dysfunction, and was authorised by the FDA and European Medicines Agency (EMA) in 1998 [94]. Studies of its use in pulmonary hypertension have been explored, led to granting marketing authorisation for pulmonary hypertension by the FDA and EMA in 2005 [94].

Sildenafil acts by preventing the conversion of cyclic guanosine monophosphate (cGMP) to GMP through the inhibition of PDE5 [94]. Higher levels of cGMP lead to the relaxation of vascular smooth muscle and pulmonary vasodilation [94]. Sildenafil may act in AD by increasing neurite growth and decreasing phospho-tau expression as seen in AD patient-induced pluripotent stem cell-derived neuron models [45]. Sildenafil phosphorylates Akt, which is associated with an increase in inhibitory GSK-3β phosphorylation, providing a plausible explanation for the reduction in tau hyperphosphorylation, which was observed in a SAMP8 model [46]. It may additionally act by improving central nervous system haemodynamic function and increasing oxygen levels, as observed in patients with AD [48]. It was found to increase the expression of β-site APP-cleaving enzyme 1 (BACE1) in an accelerated aging mouse model of dementia and reduce hippocampal Aβ42 levels, which, in turn, could mediate the parallel decline in glial fibrillary acidic protein (GFAP) expression [49]. It exerts a protective effect on neurons treated with Aβ [95]. Although in a study on a Tg2576 transgenic mouse model of AD, there was no reduction in the brain Aβ burden, sildenafil was found to reduce tau hyperphosphorylation, decrease the activity of GSK 3β (GSK3β) and cyclin-dependent kinase 5 (CDK5) (p25/p35 ratio), increase the levels of brain-derived neurotrophic factor (BDNF), and increase the activity of Arc [47]. Sildenafil decreases α-synuclein levels and oxidative stress in rats with aluminium-induced cognitive impairment [50] and rescues protein kinase B/phosphorylated cAMP response element-binding protein (PKG/pCREB) signalling in APP/PS1 transgenic mice [51]. It increased the levels of activated JNK (p-JNK (c-Jun N-terminal kinase)) found in the hippocampus of SAMP8 mice [52]) and upregulated heme oxygenase-1 in neurons challenged with advanced glycation end products (AGEs) [53]. It can also regulate nitric oxide (NO)-cGMP signalling in a mouse model of accelerated aging [54] and downregulate the expression of proapoptotic proteins in aged mice [55].

Non-clinical evidence is broadly consistent in showing the benefit of sildenafil on cognition. Sildenafil improved cognition in several mouse models of AD, including scopolamine-induced dementia [96], NO synthase N(omega)-nitro-L-arginine methyl ester (L-NAME)-induced dementia [97, 98], the APP/PS1 mouse model of AD [51, 99–101], the Tg2576 transgenic mouse model of AD [47], SAMP-8 mice [46, 49, 52], age-related AD models [102, 103] and aluminium-induced AD models [50].

On the other hand, the clinical evidence supporting the role of sildenafil in AD is limited. Only two clinical studies were found, neither of which considered cognition as an outcome. One study in twelve persons with AD reported that a single dose of sildenafil improved cerebral hemodynamic function and increased brain oxygen metabolism [48]. Another study revealed that sildenafil attenuated the increased fractional amplitude of low-frequency fluctuations in ten persons with AD [56]. There were no ongoing trials at the time of writing, although AriBio is conducting a Phase III study of PDE 5 inhibitor similar to sildenafil (NCT05531526).

Epidemiological studies present conflicting findings on the benefit of sildenafil in AD patients. Two studies using large healthcare insurance claims databases and propensity score matching/stratification revealed that sildenafil was associated with a lower risk of AD [45, 104]. A more methodologically nuanced study using Medicare health insurance data revealed that, despite the use of different analytic methods, sildenafil was not associated with a lower risk of AD [105]. Finally, a case‒control study using electronic medical records found that the use of sildenafil did not have a protective effect against AD [106]. However, the study population was unmatched, suggesting a high possibility of confounding.

#### Riluzole

Riluzole is a glutamate antagonist that is currently licenced in the US, Europe and the UK to prolong survival in individuals with amyotrophic lateral sclerosis (ALS). Its development in the 1950 s focused on its action as a centrally acting muscle relaxant, but researchers soon shifted their attention to its anticonvulsant and neuroprotective properties [107]. It was marketed in the US as early as 1995 [108]. Unlike sildenafil, it has not yet been repurposed. However, it has been studied in trials for posttraumatic stress disorder [109], depression [110], acute spinal cord injury [111] and cerebellar ataxia [112], indicating its potential to target other brain disorders.

The poly-pharmacology of riluzole indicates that it may be therapeutic under various conditions. Several mechanisms of action have been identified, including the rapid inactivation of voltage-dependent sodium channels, the inhibition of various voltage-gated K + channels and NMDA (N-methyl-D-aspartate) receptors, and the potentiation of GABA (gamma-aminobutyric acid) receptor action and glutamate uptake [107]. Riluzole may act in AD by inhibiting glutamatergic neurotransmission and inactivating voltage-dependent sodium channels, with the overall effect of preventing neuronal death by preventing excitotoxicity [57, 113]. The glutamatergic effects of riluzole appear to be effective in normalising glutamatergic neurotransmission and improving cognition [114, 115]. The normalisation of neurotransmission may also occur through the protection of neuronal hyperexcitation induced by Aβ, as observed in rat hippocampal neurons [59, 67], and through the potentiation of postsynaptic GABA receptor function, which was found to improve cognition in rats [116]. It may improve cognition by increasing the levels of BDNF in mice with doxorubicin-induced cognitive impairment [60]. Riluzole improved the cognitive impairment induced by lipopolysaccharide in mice with SLC1A1/EAAT3 (solute carrier family 1 member 1/Excitatory amino acid transporter 3) expression knocked down, suggesting that it may normalise the expression of EAAT3 [61, 62] and glucose metabolism [63]. Riluzole decreased tau levels in a mouse model of AD with tauopathy [64], attenuated hippocampal acetylcholinesterase (AChE) activity and decreased the levels of several oxidative stress markers in rats after an intrahippocampal injection of Aβ [66]. It has been associated with lower levels of disease-related microglia in a transgenic mouse model of early-onset AD [65] as well as with increased dendrite density [67] which is gradually lost in neurodegenerative disease.

Studies in mouse models of cognitive impairment that measured cognitive outcomes after riluzole treatment have consistently shown that riluzole improves cognition. Riluzole rescued cognition in several studies in which Aβ was used to induce AD-like neuropathology either directly or through specific mouse models [62–66, 114–118] and in one study in aged mice [67]. It also rescued cognition in AD-like pathology induced by scopolamine/sodium nitrite [119] and scopolamine [120].

There is limited clinical evidence regarding the benefit of riluzole in AD patients. One small 6-month clinical trial in 50 people with probable AD mini-mental state examination (MMSE) 19–27) reported that riluzole had a protective effect on brain glucose metabolism compared to placebo, with the most robust effect in posterior cingulate, and effects in precuneus, lateral temporal, right hippocampus and frontal cortex [68]. Although underpowered for statistical evaluation, there were numerical benefits on cognitive outcomes and a significant correlation between cognitive outcomes and positron emission tomography (PET) biomarkers. There is a significant evidence base of clinical trials supporting benefit of riluzole treatment in people with ALS [121], another progressive neurodegenerative disease.

### Stakeholder consultation outcomes

Stakeholder representatives reviewed the three priority candidates - herpes zoster vaccine, riluzole and sildenafil. Regarding the strength of evidence, the panel concurred that each candidate showed encouraging supporting data, with a particular strength in the scale of the herpes zoster vaccine natural science studies, the non-clinical evidence for riluzole and the mechanistic rationale for sildenafil. Side effects were raised as a moderate concern for both riluzole and sildenafil, particularly relating to the unknown effect of longer-term use of sildenafil since current use is predominantly intermittent. In the case of riluzole, concerns were raised regarding the monitoring requirements for renal side effects, although there was a consensus that this would be an acceptable inconvenience if the treatment was effective. The group felt that from a pragmatic perspective the herpes zoster vaccine had significant strengths since it required a maximum of two doses and had very well-established safety, requiring minimal monitoring post-vaccination. There was a 100% consensus that all three priority candidates were acceptable for use in the target population and should be taken forward to clinical trials. In a vote to rank the three candidates, the most popular candidate was the herpes zoster vaccine, followed by sildenafil and then riluzole.

#### Overall candidate ranking

The top three candidates emerging from the expert review and Delphi consensus were the herpes zoster vaccine, riluzole and sildenafil which all had equal priority based on the Delphi process. In the subsequent stakeholder consultation process the herpes zoster vaccine was given highest priority, followed by sildenafil and then riluzole. The overall prioritisation reflects this final ranking.

## Discussion

This study has performed an updated review and Delphi consensus to identify novel candidates for repurposing for the treatment of AD. This work identified three high-priority candidates. The Herpes zoster live attenuated vaccine is recommended for protection against shingles in the UK, US and other countries and is undergoing roll-out in defined age groups according to the national criteria for shingles risk. The glutamate antagonist riluzole is currently widely licenced as a treatment for ALS, and the PDE 5 inhibitor sildenafil is used predominantly as a treatment for erectile dysfunction but has several mechanistic effects that target neurodegenerative pathways. Lithium was one of the shortlisted compounds in the current Delphi, but was not ranked as a priority candidate. A subsequent paper highlighting a link between dementia and reduced dietary lithium does provide additional evidence to support lithium as a candidate, and lithium does therefore merit some further consideration [122].

Each of the priority candidates has evidence supporting relevant underlying mechanisms of action, non-clinical studies and clinical evidence from epidemiological studies and/or preliminary clinical trials. The tolerability of each of these compounds is also suitable for administration to a frailer population of older individuals as part of a well-monitored clinical trial programme.

We therefore recommend each of these therapeutic approaches as a high priority for clinical trials for the treatment or prevention of AD. There are however important differences in the potential benefits with the different treatment approaches. Treatment with the herpes zoster vaccine has the potential advantage of having a wide-reaching impact, which, if confirmed in a clinical trial, could confer a substantial benefit at the population level for prevention. In contrast, sildenafil and riluzole have potential utility for further investigation as disease modifying treatments for people with established clinical or pre-clinical Alzheimer’s disease, and possibly also for people with concurrent cerebrovascular disease in the case of sildenafil.

Given the low safety risk profile of these candidates, they would be suitable for assessment through remote or hybrid trial designs. The PROTECT platform, which supports international cohorts in the UK, Norway and Canada, offers a well-established means of delivering trials via this approach. PROTECT coordinates existing cohorts of well-characterised participants, currently with over 40,000 active participants worldwide, and a suite of computerised neuropsychology assessments that are validated for trial use. The combination of repurposing and efficient trial design raises the potential to fast-track multiple drug candidates through trials in an affordable way and to ensure that new treatments reach patients faster.

The strength of the current Delphi is the rich breadth of clinical, scientific and drug discovery expertise. The main limitation is that it is dependent on candidates that investigators have already studied, and potentially could exclude candidates that could be identified through high-throughput screening. It is however one important tool to highlight the important high priority candidates that need to be investigated in a clinical trial programme.

## Supplementary Information

Supplementary Material 1.

## Data Availability

The datasets used and/or analysed during the current study are available from the corresponding author on reasonable request.
