# Supplementary material for: Drug repurposing for Alzheimer’s disease: a Delphi consensus and stakeholder consultation
Source: Alzheimers Res Ther. 2025 Nov 18;17:237. doi: 10.1186/s13195-025-01895-4 (PMC12625010; doi:10.1186/s13195-025-01895-4)
Supplement: Supplementary file 1 — Supplementary Material 1. [file 13195_2025_1895_MOESM1_ESM.pdf]

Candidate	Nominations	Action	proposed by:	drug, chemical compound,	Current drug purpose	Associated drugs	Group
riluzole	4		HF, JC, AC, CB	chemical compound	amyotrophic lateral sclerosis & motor neuron diseases	sodium channel blockers	1
fingolimod	4		JC, ZI, RL, ML	chemical compound	multiple sclerosis	siponimod	1
sildenafil	4		JC, AC, CB, SG	drug	erectile dysfunction & pulmonary hypert	tadafil	3
cytisine	3		GW, DA, RK	chemical compound	smoking cessation	vareniciline	2
vortoxetine	3		DA, AC, CB	chemical compound	depression	serotonin reuptake inhibitor class	5
lithium	3		SL, DA, SG	drug	mood stabiliser	Mood stabilizers	5
cannabinoid (CBD)	3		JM, DA, RL	chemical compound	used to treat seizures	THC	7
vacyclovir/ valacyclovir/valaciclovir	3	exclude - already in trial - note that is has been flagged	DA, AC, CB	chemical compound	antiviral drug - herpes	famciclovir	19
dasatinib	3		MsM, ML and ???	chemical compound	leukaemia	imatinib, nilotinib,	
tabex	2		GW, RK	supplement	smoking cessation	cytisine	2
etanercept	2		HF, CR	drug	rheumatoid arthritis (RA)& other autoimmune diseases		6
pioglitazone	2		DA, JS	chemical compound	type II diabetes	Rosiglitazone	14
nicotine patches	1		JM	drug	smoking cessation	vareniciline, nicotine gums	2
candesartan	1		JC	drug	hypertension, heart failure	losartan, valsartan	3
Atenolol	1		JS	drug	hypertension, arrhythmia	metaprolol, propanolol, bisoprolol	3
prazosin	1		JM	drug	antihypertensive	doxazosin, terazosin	3
monophopshoryl lipid A (TLR4 antagonist).	1		WN	vaccine?	antiviral?	other TLR agonists	4
Paxlovid	1		RM	drug	antiviral drug	Lopinar/ritonavir, darunavir	4
citalopram	1		JM	drug	depression	serotonin reuptake inhibitor class	5
escitalopram	1		JM	drug	depression	citalopram, sertraline, fluvoxamine	5
GSK1482160A (P2X7R antagonist)	1		WN	chemical compound	depression? Neuroinflammation?	AZD9056, CE-224,535	5
imipramine	1		RL	drug	antidepressant, bedwetting	amitriptyline, nortriptyline, desipramin	5
rolipram	1		RL	chemical compound	Anti depressant	roflumilas, cilomilast	5
adalimunam	1		CR	drug	monoclonal antibody used for RA	infliximab, golimumab	6
infliximab	1		CR	drug	monoclonal antibody used for autoimmune disease (Crohn's disease, RA, ulcerative colitis	adalimumab, etanercept	6
Levitaracetam	1		TF	chemical compound	epilepsy	anticonvulsants	7
diclofenac	1		DA	chemical compound	anti-inflammatory (gout)	ibuprofen, naproxen	8
glucocorticoid-induced leucine zipper (GILZ) analogue (GA)	1		WN		anti inflammatory?	dexamethasone, prednisolone	8
plant-based cholinesterase inhibitors that have additional anti-inflammatory action	1		ZI	chemical compounds	anti inflammatory	colchicine, berberine	8
prednisone	1		HF	chemical compound	anti inflammatory, asthma, COPD	dexamethasone, hydrocortisone	8
reparixin (CXCR2 antagonist)	1		WN	drug	anti inflammatory	navarixin	8
Febuxostat	1		JS	chemical compound	prevents gout	allopurinal	8
Armodafinil	1		TF	chemical compound	wakefulness/ sleep disorders	modafinil	9
clemasine/ clemastine	1		RL	chemical compound	antihistamine, sedative side effects	meclastin	9
Melatonin / circadian rhythm balancing	1		RM	hormone	insominia	ramelteon, tasimelteon, agomelatine	9
berberine (NFkappaB antagonists)	1		WN	chemical compound	anticancer role	curcumin, resveratrol, withaferin A	10
bexarotene	1		ZI	chemical compound	anticancer role	alitretinoin, isotretinoin	10
bicalutimide	1		ZI	chemical compound	anticancer role - prostate cancer	enzalutamide, flutamide, apalutamide	10
gleevec (Imatinib)	1		RL	drug (chemical compoun	cancer	dasatinib, nilotinib, bosutinib	10
Lonafarnib	1		JC	chemical compound	Cancer & Hutchinson-Gilford progeria sy	tipifarnib	10
pomalidomide (IMiD)	1		NG	chemical compound	anti-cancer	lenalidomide, thalidomide	10
TPCA-1	1		WN		cancer	Bay 11-7082, MLN120B	10
beta-carotene	1		ZI	Chemical compound, die	antioxidant	vitamin A, retinol, lutein, lycopene	11
circumin/ curcumin	1		ZI	chemical compound	dietary supplement	demethoxycurcumin, bisdemethoxcurc	11
coffee	1		ZI	diet supplement	antioxidant	green tea extract, theobromine	11
green tea	1		SG	diet supplement	antioxidant	epigallocatechin gallate	11
hibiscus	1		ZI	diet supplement	antihpertensive, antioxidant	roselle extract, anthocyanins	11
pecan extract	1		ZI	diet supplement	antioxidant, anti-inflammatory	ellagic acid, quercetin, polyphenols	11
resveratrol	1		ZI	diet supplement?	anticancer/ anti inflammatory - no evide	pterostilbene	11
donepezil	1		RL	chemical compound	dementia	galantamine, rivastigmine	12
rivastigmine transdermal patch	1		RL	chemical compound	alzheimers disease	donepezil galantamine	12
gemfibrazole/gemfibrozil	1		JC	drug	hyperlipidaemia, abnormal lipid levels	fenofibrate, bezafibrate	13
metformin	1		DA	chemical compound	type II diabetes	phenformin	14
mounjaro (tirzepatide)	1		NG	drug	type II diabetes	semaglutide, dulaglutide, liraglutide	14
sitagliptin (or another widely used gliptin)	1		NG	drug	type II diabetes	linaglipitin	14
tirzepatide (diabetic dose)	1		RM	chemical compound	type II diabetes	semaglutide, dulaglutide, liraglutide	14
Methylphenidate	1		JM	chemical compound	ADHD	dexmethylphenidate, amphetamine sal	15
rasagiline	1		JC	chemical compound	parkinson's disease	selegiline	16
rotigotine/rosigitine	1		JC	chemical compound	parkinson's disease	pramipexole, ropinirole, bromocriptine	16
selegiline (for NPS)	1		JC	chemical compound	parkinson's disease	rasagiline, safinamide	16
risperidone (& other neuroleptics)	1		JM	chemical compound	antipyschotic	obeticholic acid	17
olanzapine	1		RL	drug	antipyschotic	risperidone, clozapine, quetiapine	17
Ursodeoxycholic acid (multiple targets - rescues ER-mito interactions)	1		WN	chemical compound	liver disease	obeticholic acid, chenodeoxycholic acid	18
phenserine	1	exclude - already in trial - note that is has been flagged	DA	drug	alzheimers disease	donepezil, rivastigmine	19
semaglutide (diabetic dose)	1	exclude - already in trial - note that is has been flagged	RM	drug	type II diabetes	liraglutide	19
fasudil	1	exclude - already in trial - note that is has been flagged	DA	drug (chemical compoun	Rho kinase inhibitor	ripasudil	19
tetrahydrocannabinol (THC)	1		JM	chemical compound	Pain, Muscle spasticity, Glaucoma, Insomnia, Low appetite, Nausea, Anxiety	cannabidiol, dronabinol	20
Anti Il6 monoclonals (anti-TNF alpha)	1		HF	drug	used forinflammatory diseases & many c	tocilizumab, sarilumab	8, 10
diabetes treatments	1		JM	drug	Glycemic control	metformin, semaglutide	
RA autoimmune drugs (methotrexate)	1		HF	drug	autoimmune diseases	leflunomide, azathiopind	
relevant cancer drugs	1		HF	drug	anticancer agents	cisplatine, checkpoint inhibitiors	
nanolithium	1	exclude - already in trial - note that is has been flagged	MsM	chemical compound	mood stabilizer	lithium carbonate	
RAPAMYCIN (sirolimus)	1		FW	drug	immunosuppressant	everolimus, temsirolimus	
Tacrolimus (FK506)	1		FW	drug	immune supressant	cyclosporine, pimecrolimus	
Temsirolimus	1		FW	drug	mTOR inhibitor	everolimus, sirolimus	
Quercetin	1		MsM	chemical compound	antioxidant, anti-inflammatory	fisetin, kaempferol	
Zidovudine	1		ML	drug	HIV/AIDS	lamivudine	
Andrographolide	1		ML	chemical compound	anti inflammatory	artesunate	
Ergothioneine	1		ML	chemical compound	antioxidant amino acid	glutathione	
Lysoveta	1		ML	drug, chemical compoun	experimental	Omega-3 fatty acids	
